# Alberta family physicians’ willingness to work during an influenza pandemic: a cross-sectional study

**DOI:** 10.1186/1447-056X-12-3

**Published:** 2013-06-26

**Authors:** James A Dickinson, Gisoo Bani-Adam, Tyler Williamson, Sandy Berzins, Craig Pearce, Leah Ricketson, Emily Medd

**Affiliations:** 1 Department of Family Medicine, Research Office, G012, Health Sciences Centre, 3330 Hospital Drive NW, Calgary Alberta T2N 4N1, Canada; 2 Centre for Studies in Primary Care, Queen’s University, 220 Bagot Street, P.O. Bag 8888, Kingston, Ontario K7L 5E9, Canada; 3 Department of Community Health Sciences, Faculty of Medicine, 3rd Floor TRW Building, 3280 Hospital Drive NW, Calgary, Alberta T2N 4Z6, Canada; 4 Department of Pediatrics, Alberta Childrens Hospital, 2888 Shaganappi Trail NW, Calgary T3B 6A8, Canada

**Keywords:** Physicians, Family, Occupational health, Pandemic influenza A H1N1 Virus

## Abstract

**Objective:**

Effective pandemic responses rely on frontline healthcare workers continuing to work despite increased risk to themselves. Our objective was to investigate Alberta family physicians willingness to work during an influenza pandemic. Design: Cross-sectional survey. Setting: Alberta prior to the fall wave of the H1N1 epidemic. Participants: 192 participants from a random sample of 1000 Alberta family physicians stratified by region. Main Outcome Measures: Willingness to work through difficult scenarios created by an influenza epidemic.

**Results:**

The corrected response rate was 22%. The most physicians who responded were willing to continue working through some scenarios caused by a pandemic, but in other circumstances less than 50% would continue. Men were more willing to continue working than women. In some situations South African and British trained physicians were more willing to continue working than other groups.

**Conclusions:**

Although many physicians intend to maintain their practices in the event of a pandemic, in some circumstances fewer are willing to work. Pandemic preparation requires ensuring a workforce is available. Healthcare systems must provide frontline healthcare workers with the support and resources they need to enable them to continue providing care.

## Background

The pandemic H1N1 virus first emerged in April 2009 and rapidly spread throughout the world [1]. Initial overload of intensive care units caused alarm, though subsequently the case-fatality rate of this strain appeared to be around 0.5%, near the upper range for seasonal influenza [2].

Pandemics can increase morbidity and mortality, producing strain on healthcare systems and healthcare workers, including family physicians providing frontline care. Ethicists posit that frontline healthcare workers have a duty to continue working through an epidemic despite increased risk [3,4]. During the severe acute respiratory syndrome (SARS) outbreak of 2003, some doctors provided care to patients despite increased risk to themselves and their families, while others refused [5]. In Toronto, 37.5% of primary care physicians reported that they closed their clinic during the SARS epidemic [6], suggesting that primary healthcare availability suffers during times of increased occupational risk and service demand.

The Canadian Pandemic Plan suggests that during a severe pandemic, family physicians may be required to work in triage centers where they would make decisions about which patients to treat [3]. The plan also suggests that family physicians may be required to take on work that they were not trained for, such as managing ventilated patients [3]. A severe pandemic may also cause colleagues and physicians’ families to sicken or die, and disruption of services such as childcare or transport (which would lead to difficulty with staff and supplies). These scenarios challenge physicians’ duty to care. We designed a survey to understand the limits of the conditions in which Alberta physicians would be willing to work during a pandemic. The objective of the study was to explore Alberta family physicians’ experiences during the first wave of the H1N1 (swine flu) outbreak and their plans for the future. We hypothesized that there would be differences between male and female doctors, rural and urban, older and younger, and those educated in different countries. Since family physicians’ work is substantially different in rural compared to urban areas, we tested the hypothesis that responses and attitudes vary according to rurality. There had been an continuing medical education program on pandemic planning offered in Calgary two years prior that was not offered in Edmonton [7], so we also hypothesized that there would be differences between the physician groups in these otherwise similar cities.

## Methods

### Study sample

A list of all Alberta family physicians was obtained from the College of Physicians and Surgeons of Alberta (N = 3558). One thousand family physicians were identified by stratified random sampling from this registration list (250 from Edmonton, 250 from Calgary, 250 from other urban centres and 250 from rural areas. Definitions of each of these areas are detailed in the Appendix 1). Exclusion criteria were if the physician was not in primary care practice or if they worked only in academia, were no longer working in Alberta, or were not currently working for other reasons such as being retired, on vacation or personal health reasons. 122 physicians were therefore excluded. By November 5, 2009 we had received 192 completed surveys from eligible physicians, for an overall response rate of 22%.

### Survey development

A questionnaire was designed then pilot tested on a convenient group of physician colleagues who were not in the sample. Questions on physician attitude regarding working during an H1N1 pandemic were included from a recent Ontario study (personal communication, Ross Upshur, September 2009). The final questionnaire, which included both closed- and open-ended questions, was sent to selected family physicians after the summer vacation, and before the fall influenza season began.

### Survey process

Selected physicians were provided with up to 3 packages sent 2 weeks apart: on September 1, 14 and 30, 2009 respectively. The first and third packages included an information sheet, consent form, copy of the questionnaire, and a negative response sheet. The second package included an information sheet with a request to access the survey on the Department of Family Medicine website and a negative response sheet.

Written consent was obtained from those who agreed to participate in the study. Ethics approval for this project was given by the Conjoint Health Research Ethics Board of the Faculties of Medicine, University of Calgary on August 14, 2009. The study was endorsed by the Alberta Medical Association (AMA) and the College of Physicians and Surgeons of Alberta, and these endorsements were mentioned in the cover letter.

### Statistical analysis

Physicians were grouped, where possible, into countries that share a similar style of medical education. Specifically, North America (Canada and United States of America), South Africa, and British (United Kingdom, Ireland, and Australia). All remaining countries had insufficient representation and were grouped together as other (Table 1). Age was reported as a categorical variable as that is how the information was obtained in the questionnaire (age groups 25 to 34, 35 to 44, 45 to 54, 55 to 64, and 65 + yrs).

**Table 1:** Characteristics of responding physicians compared to the total number of registered practicing family physicians in each region

	**Calgary**		**Edmonton**		**Other urban**		**Rural**		**Total**	
	**Survey N (%)**	**Provincial data N (%)**	**Survey N (%)**	**Provincial data N (%)**	**Survey N (%)**	**Provincial data N (%)**	**Survey N (%)**	**Provincial data N (%)**	**Survey N**	**Provincial data N**
**Sex**										
Male	31 (53.4)	630 (50.0)	17 (45.9)	645 (58.5)	34 (77.3)	34 (77.3)	35 (66.0)	612 (76.0)	117 (61.0)	2145 (59.2)
Female	27 (46.6)	630 (50.0)	20 (54.1)	458 (41.5)	10 (22.7)	131 (33.7)	18 (34.0)	193 (24.0)	75 (39.1)	1412 (39.0)
**Total**	58	1260	37	1103	44	389	53	805	192	3557
**Age Group**										
25-34 years	5 (8.6)	157 (11.9)	5 (13.5)	142 (12.3)	6 (13.6)	53 (12.1)	7 (13.2)	131 (18.4)	23 (12.0)	483 (13.3)
35-44 years	483 (13.3)	355 (27.0)	4 (10.8)	299 (25.8)	10 (22.7)	10 (22.7)	17 (32.1)	184 (25.8)	49 (25.5)	959 (26.5)
45-54 years	19 (32.8)	411 (31.2)	13 (35.1)	313 (27.1)	15 (34.1)	125 (28.5)	10 (18.9)	178 (25.0)	57 (29.7)	1027 (28.3)
55-64 years	12 (20.7)	281 (21.2)	11 (29.7)	277 (23.9)	9 (20.5)	98 (22.3)	14 (26.4)	154 (21.6)	46 (24.0)	810 (22.3)
65+ years	3 (5.1)	113 (8.6)	4 (10.8)	126 (10.9)	4 (9.1)	42 (9.6)	3 (5.7)	65 (9.1)	14 (7.3)	346 (9.5)
Not known	1 (1.7)		0 (0.0)		0 (0.0)		2 (3.8)		3 (1.6)	
**Total**	58	1317	37	1157	44	439	53	713	192	3625
**Country of Primary Medical Education**										
North America	40 (69.0)	908 (70.0)	25 (67.5)	816 (71.1)	28 (63.6)	276 (63.7)	30 (56.6)*	293 (40.3)	123 (64.0)	2293 (63.3)
South Africa	2 (3.5)	109 (10.0)	1 (2.7)	36 (3.1)	8 (18.2)	75 (17.3)	15 (28.3)	266 (36.6)	26 (13.5)	486 (13.4)
UK, Ireland, Australia	6 (10.3)	86 (10.0)	4 (10.8)	65 (5.6)	1 (2.3)	24 (5.5)	4 (7.5)	72 (9.9)	15 (7.8)	247 (6.8)
Other Countries	8 (13.8)	214 (20.0)	6 (16.2)	231 (20.1)	6 (13.6)	58 (13.4)	4 (7.5)	96 (13.2)	24 (12.5)	599 (16.5)
Not known	2 (3.5)		1 (2.7)		1 (2.3)		0 (0.0)		4 (2.1)	
**Total**	58	214 (20.0)	37	1148	44	433	53	727	192	3625

Primary data analysis included frequencies, cross tabulations, and chi-square tests to examine the association between willingness to work and physician demographics (i.e. gender, physician age group, country of primary education, and regional location of primary practice). A secondary, exploratory, analysis examined the effect of a response of “likely” willing to work and the potential synergistic effects of physician demographics, using multivariable logistic regression. Model selection was founded on epidemiological constructs using a backward elimination strategy. Possible interaction effects were first examined, where sufficient data was available, and then simpler additive effects were considered. Categorical variables were modeled with the most prevalent category as the baseline. Whenever a single level within a category appeared statistically important, the other levels of the category were tested for differences from the baseline and if not different, included in the baseline group. A significance level of 0.05 was used throughout this process. All analyses were done using STATA statistical software version 11.1.

## Results

The largest representation came from the 45–54 year old group. There were more male than female physicians outside the major cities (Table 1). Males had a higher response rate in all regions.

To examine the representativeness of the survey respondents, the gender distribution by region of primary practice of those responding was compared to the gender distribution obtained from the list of family physicians from the College of Physicians and Surgeons of Alberta using a two-sample proportion test. There were no statistically significant differences in gender distribution. Age group distribution and country of primary medical education were also compared; the only statistically significant difference was found in the higher proportion of North American trained rural physicians among the respondents (56.6%) compared to the provincial distribution (Table 1).

We asked physicians about their concern regarding infection by pandemic H1N1. More than half of the survey participants (64%) were worried or very worried about being infected by H1N1 due to the nature of their occupation, while 36% were not worried. Most respondents (77%) also indicated that they were worried or very worried about bringing an infection home to their family, 23% were not worried.

Thirty-four percent of Calgary physicians indicated their work had already been affected by H1N1, while fewer physicians from Edmonton (17%), other urban (14%) and rural (11%) reported that they were affected by the first wave of H1N1 (Chi-squared (3df) = 10.99; p = 0.012). In response to an open-ended question about how they were affected, several physicians commented on increased hand-washing due to concerns about infection, and working longer due to increased service demand.

### Willingness to work

**Figure 1:**
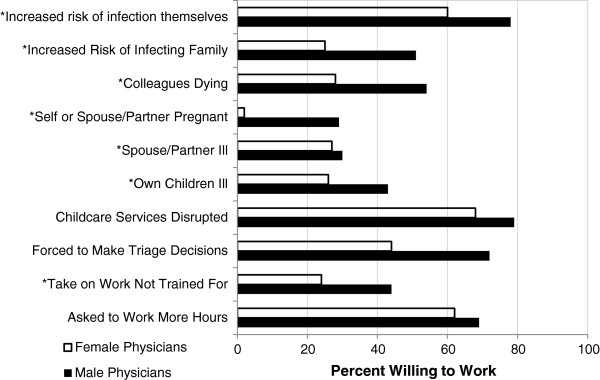
**Family physicians willingness to work in defined pandemic scenarios, by gender.** (Sum of physicians answering “likely” to work in the following circumstances). (*indicates statistically significant differences between males and females, chi square, p < 0.05.).

**Figure 2:**
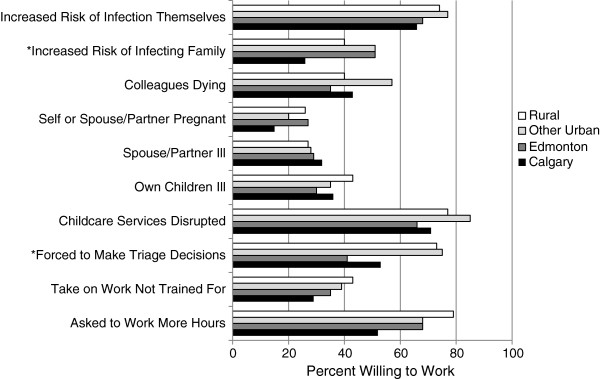
**Family physicians willingness to work in defined pandemic scenarios, by region.** (Sum of physicians answering “likely” to work in the following circumstances). (*indicates statistically significant differences between regional responses, chi square, p < 0.05).

We asked how physicians might react if a pandemic caused increased risk of infection to themselves or their family, and whether they would be willing to continue work as suggested in pandemic plans. Responses are shown in Figure 1 stratified by gender, and Figure 2 by region. Male physicians were more likely than female physicians to report that they would continue working in each of these situations, but it is notable that less than half of male doctors would continue to work if their family were affected, or if they were asked to take on work that they were not trained for. Differences between regions were small: only three of the regional differences were significant. Physicians from rural and other urban areas were significantly more likely to respond that they would continue working if there was a disruption to transportation (p = 0.005) compared to physicians from the major cities. Similarly, rural and other urban physicians were more likely than Calgary and Edmonton physicians to respond that they were willing to work if asked to make decisions about not treating certain patients due to resource constraints (p = 0.002). Physicians from Edmonton and other urban areas were significantly more likely than physicians from Calgary to assert they would be willing to work despite a greater risk than usual of infecting their family at home (p = 0.02).

The logistic regression approach demonstrated that after adjusting for possible confounding effects of age, female physicians were less willing to work in many of the proposed situations, especially where there is a risk or need to care for their family (Table 2). There were also differences in responses according to the origin of doctors. Physicians trained in South Africa and British style medical schools expressed greater willingness to continue work than others. Rural physicians expressed that they were more willing to work extra hours and if there was a shortage of fuel, leading to a disruption of transportation. In other proposed scenarios, no significant differences were found between physician groups. The age of physician respondents did not play a significant role in any regression model tested.

**Table 2:** Logistic regression models of physicians’ anticipated willingness to work in various scenarios

**Scenario**	**Predictor**	**OR**	**Std. Error**	**Z**	**P > |z|**	**95% Conf. Interval**
If there was a greater than usual risk of infecting your family at home	Female	0.30	0.10	−3.51	0.000	0.15 – 0.59
	British	5.07	3.20	2.57	0.010	1.47 – 17.49
If you were asked to take on different or additional work/duties for which you have not been trained	Female	0.45	0.15	−2.40	0.016	0.23 – 0.86
	Sth Afr	2.42	1.06	2.01	0.044	1.02 – 5.70
If you were asked to work more hours	Rural	2.35	0.90	2.24	0.025	1.11 – 4.97
If your children fell ill	Female	0.46	0.17	−2.10	0.036	0.22 – 0.95
If you or your spouse/partner were pregnant	Female	0.16	0.13	−2.32	0.02	0.035 – 0.75
If your colleagues were dying	Female	0.34	0.11	−3.22	0.001	0.18 – 0.66
	Sth Afr	2.44	1.11	1.97	0.049	1.00 – 5.97
If there was shortage of fuel, leading to disruption of transport (private or public)	Female	0.37	0.12	−3.11	0.002	0.20 – 0.70
	Rural	2.45	0.87	2.52	0.012	1.22 – 4.92
If you had to make decisions about not treating certain patients because of resource constraints	Female	0.32	0.10	−3.65	0.000	0.17 – 0.59

Odds ratio above 1 signifies that doctors with the condition are more likely to keep working, lower than 1 less likely to keep working.

*Female* = compared to males.

*Sth Afr* = physicians who completed primary medical education in South Africa compared to all other countries.

*British* = physicians who completed primary medical education in Australia, Ireland and The United Kingdom compared to all other countries.

*Rural* = Physicians working in rural locations compared to all other physicians in survey.

Note: There was no significant effect on response by sex, age, location of practice, or country of primary medical education for the remaining scenarios:

• If there was a greater than usual risk of becoming infected at work and falling ill yourself.

• If schools/nurseries were closed or other childcare services were disrupted.

• If your spouse/partner fell ill.

• If you were asked to work at a different hospital/healthcare practice from normal.

• If you had to work with untrained volunteers or workers brought out of retirement.

Fortuitously, the first wave of the epidemic occurred in May and June, then died off during the summer, and returned in late October. Thus most of our survey responses came in while there was uncertainty about how severe the H1N1 2009 pandemic would become, and concern remained about the severe cases that had filled intensive care units. Despite that, more than half the physicians (78% of males, 60% of females) who responded to our survey said they would be willing to continue working despite increased risk to themselves. Alberta would not be without family physicians in the event of a severe pandemic; however, care would be compromised due to fewer physicians being available at a time of increased service demand. If physicians’ family members were ill, fewer respondents reported being willing to work, and there was also unwillingness to work in several situations suggested by the pandemic plans.

## Discussion

These results suggest that during a severe influenza outbreak more than half of responding Alberta physicians may be available and willing to work; however, in a severe pandemic, these numbers may drop due to their own illness, or unforeseen circumstances. Men appear to be more willing to continue working than women. There may be differences according to country of origin, due perhaps to different cultural acceptance of risk or perception of duty.

### Limitations

The corrected response rate for this survey (22%) was low despite sending three letters in sequence; therefore, there may be response bias, and we cannot know in what direction this would affect the results. Because of the ethics committee ruling, we were unable to contact a sample of non-responders for a comparison with responders. However, the gender composition of the responding group reflects the demographic features of the primary care physician population in Alberta, and sample size is adequate to demonstrate clear differences in gender responses, so these findings likely reflect reality (Table 1). Sample sizes are small for differences between physicians from different origins, and for urban/rural differences, so power to demonstrate difference is limited and small biases due to differential response rates could affect the findings substantially. Further, we performed multiple comparisons, so if a correction for multiplicity was applied, some differences would be regarded as non-significant. Conclusions from comparisons between the sub-groups thus reflect the opinions of the survey participants and potentially not the views of the family physician population overall, so should therefore be viewed with caution.

Despite these limitations, there is a degree of consistency among the responses that raises concerns about physicians’ willingness to work during a pandemic. Some writers on this topic assert that physicians and other healthcare workers have moral, ethical, and legal obligations to continue working despite increased risk to themselves and their families [8], though others point out that most health care workers did not volunteer to be “heros” [5]. In Canada, physicians are expected to adhere to the *Code of Ethics* published by the CMA [9]. These guidelines do not provide definitive directions to physicians regarding whether to work during a pandemic. Each province has its own statute that can conscript physicians to treat patients during a declared emergency. Alberta has the *Emergency Management Act*, but there has been minimal recent public or professional discussion about this act and its implications [4]. Though the law could compel doctors to work, it is unlikely to be effective, and any legal action would likely not commence until well after the need were past.

These are only answers to a questionnaire about a hypothetical, though nonetheless imminently possible threat at the time it was distributed. It is impossible to know what would actually happen until faced with the real crisis: doctors might indeed stay at their posts to a greater extent than they answer in this questionnaire, or they might have over-stated their likelihood of working. Previous studies worldwide have also explored the willingness of physicians to work during a severe pandemic, and found similar responses. Not all healthcare workers willingly accept the increased risk associated with their profession, but in a German study, physicians were more willing to work than those who were not in direct clinical care [10]. A qualitative study in the United Kingdom found that most physicians felt an obligation to work during a pandemic, though there were barriers to their willingness and ability to work [11]. Fear of infecting their families was a common concern, as well as barriers to finding childcare in order to continue working [11,12]. Barriers to obtaining personal protective equipment (PPE) [12], and concerns about contracting influenza caused some reluctance [13]. Fear of social ostracism for themselves and their families was also a concern for physicians in Singapore [13]; such events occurred in the SARS epidemic in Toronto [14], and may have affected Canadian physicians’ responses in our sample. Ultimately the only valid test of actions is to see what happens when an epidemic occurs: but these findings correspond with the limited data available in recent epidemics [5,6].

## Conclusions

These findings have implications for health care planning policy development. Our results suggest that during an outbreak more than half of Alberta physicians will be available and willing to work; however, in the midst of a severe pandemic, these numbers may drop due to their own illness, or unforeseen circumstances. Men appear to be more willing to continue working than women. There may be differences according to country of origin, due perhaps to different cultural acceptance of risk or perception of duty.

Governments have an obligation to assist healthcare workers in providing service during an epidemic [8]. This includes decreasing the risk for healthcare workers as much as possible through providing education, information, protective equipment, and chemoprophylaxis or vaccination [8]. Female physicians especially need to be provided with support in such aspects as childcare and protective measures to encourage them to maintain frontline care. This will become more important as women become the majority of doctors.

We recognize that our findings come from only one province, and a survey with a low response rate and small sample size especially for subgroup analyses. Therefore, we suggest the need for further research to better understand these issues.

## Appendix 1

### **Definitions of strata for cities and rural areas.**

Calgary: the city of Calgary including the surrounding areas of Airdrie, Bragg Creek, Chestermere, Cochrane, De Winton, Okotoks, Redwood Meadows, and Siksika reserve. N of doctors:1317

Edmonton: the city of Edmonton including surrounding areas of Ardrossan, Bashaw, Beaumont, Calmar, Devon, Fort Saskatchewan, Gibbons, Sherwood Park, Spruce Grove, and St. Albert. N of doctors:1148

Other Urban centers: Banff, Canmore, Fort MacMurray, Grand Prairie, Leduc, Lethbridge, Medicine Hat and Red Deer. N of doctors:433

Rural Alberta: All other areas of Alberta. N of doctors:727

## Competing interests

The authors declare that they have no competing interests.

## Authors’ contributions

JD had the original idea for the research, and directs the TARRANT influenza surveillance program. GBA was the primary developer of the survey, SB, LR, CP and EM worked in the TARRANT program: they checked data, conducted analyses, and developed graphs. TW performed the statistical analyses. All authors contributed to planning, writing, and revising the paper. JD is the guarantor and corresponding author. All authors read and approved the final manuscript.

## Authors’ information

(At the time of article writing)

JD is a Professor of Family Medicine and of Community Health Sciences, University of Calgary and the director of the TARRANT Influenza Surveillance System in Alberta.

GBA is an international trained medical doctor and was the study coordinator.

SB was a PhD student in Epidemiology, Dept. of Community Health Sciences, University of Calgary, and the coordinator of TARRANT Influenza Surveillance System.

CP was an MSc student in healthcare epidemiology, Dept. of Community Health Sciences, University of Calgary and a research assistant for the TARRANT Influenza Surveillance System.

LR was an MSc student in healthcare epidemiology, Dept. of Community Health Sciences, University of Calgary and a research assistant for the TARRANT Influenza Surveillance System.

TW was a PhD student in Biostatistics, Dept. of Community Health Sciences, University of Calgary, and the biostatistician for the research hub in the Family Medicine Department at the University of Calgary.

EM completed her Master of Science in Epidemiology at the University of Calgary and currently works as the senior data analyst for the HIV and Hepatitis C Prevention Research team at the University of Ottawa. She is a former research assistant for the TARRANT Influenza Surveillance System.
